# Supplementary material for: Immunopathology of Pulmonary Mycobacterium tuberculosis Infection in a Humanized Mouse Model
Source: Int J Mol Sci. 2024 Jan 29;25(3):1656. doi: 10.3390/ijms25031656 (PMC10855034; doi:10.3390/ijms25031656)
Supplement: Supplementary file 1 [file ijms-25-01656-s001.zip › ijms-2831437-supplementary.pdf]

Supplementary Materials

Immunopathology of Pulmonary *Mycobacterium tuberculosis* Infection in a Humanized Mouse Model

Afsal Kolloli, Ranjeet Kumar, Vishwanath Venketaraman and Selvakumar Subbian

Supplementary Figure S1.

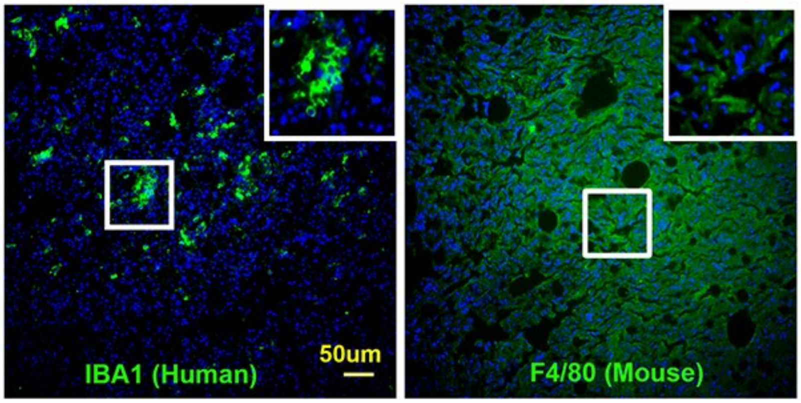

Figure S1. Validation of humanized mice using spatial expression analysis of macrophages. Antibodies specific to human (IBA-1) or mouse (F4/80) macrophages were used to confirm the presence of predominantly human cells in the lungs of humanized mice used in this study. The boxed region is magnified and shown in square insets at a higher magnification to indicate the respective antibody staining pattern**.** Image show is the lungs of infected humanized mice at 4 weeks post infection. The scale bar (50μm) is common to both the panels.

Supplementary Figure S2.

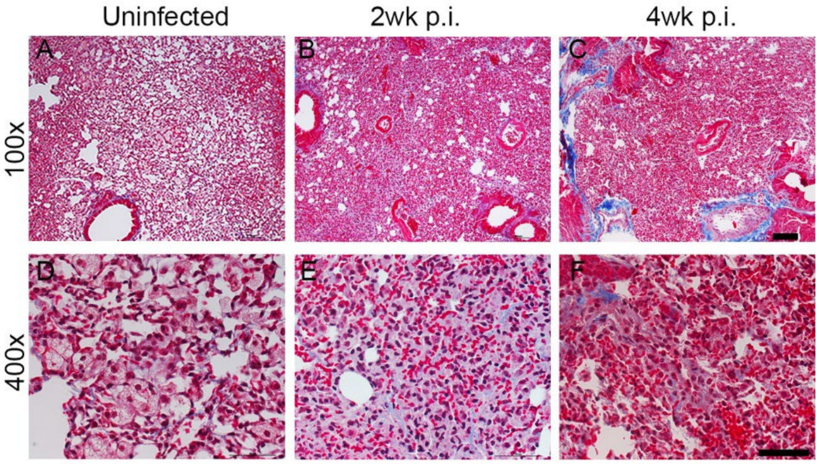

Figure S2. Histopathological analysis of collagen deposition and tissue remodeling in the lungs of humanized mouse with or without Mtb infection. **A&D.** Representative Trichrome stained image of lung section from uninfected

humanized mice. Lung parenchyma with no collagen deposition or fibrosis can be seen in the uninfected mouse lungs. **B&E.** Representative Trichrome stained image of lung section from Mtb infected humanized mice at 2 weeks post infection. At this timepoint, mild level of collagen deposition and fibrosis (blue coloration) can be seen. **C&F.** Representative Trichrome stained image of lung section from Mtb infected humanized mice at 4 weeks post infection. Prominent collagen deposition and fibrosis (blue coloration) can be seen at this time point. Images **A-C** are shown at 100x magnification and **D-F** are shown at 400x magnification. The scale bar in **A** (100μm) is common for **A-C** and the scale bar in **F** (50μm) is common for **D-F**.

Supplementary Figure S3.

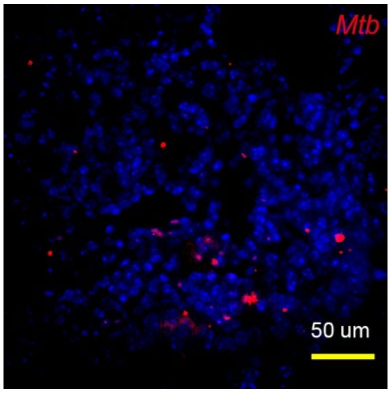

Figure S3. Visualization of Mtb (red spots) in the lungs of infected humanized mice at 4 weeks post infection. The scale bar is 50μm.
